# Wills of Medical Men

**Published:** 1921-01-01

**Authors:** 


### Wills of Medical Men.

LIEUT.-COLONEL JOHN SINCLAIR WHITE, C.B.E., M.D., of Dunluce, Bournemouth, for many years one of the best-known medical practitioners in Sheffield, who died on August 8, left estate of the value of £49,194 gross and £48,537 net. The ultimate residue is left to the Royal Infirmary, Sheffield, for a home of rest for the nurses of that institution.

SIR JOHN MCCALL, M.D., of Nevern Mansions, Earl's Court, for ten years Agent-General in London for Tasmania, left property valued at £15,307.

MR. WILLIAM NUTTALL, M.R.C.S., of Hawshead Street, Southport, Lancs, late of Bury, has left estate valued at £29,564.

DR. EUGENE TULK-HART, M.D., of Preston Park Avenue, formerly of Dyke Road, Brighton, has left £18,300.

MR. R. T. C. ROBERTSON, M.B., L.R.C.S., of Hamilton, Scotland, has left estate valued at £3,254.

MR. FRANCIS CRESSWELL, M.R.C.S., of the Poplars, Barrow-on-Soar, Leicestershire, has left £5,326.

MR. C. B. GRATTE, M.R.C.S., L.R.C.P., of Newport, Mon., hon. physician to the Newport and Monmouth County Hospital, has left £6,094.

SURGEON-MAJOR JOHN LEWTAS, C.B., Indian Medical Service, has left estate valued at £42,667.

DR. J. N. DAVIES, of Tir Caradoc, Port Talbot, left estate valued at £28,081. He left £100 to his coachman and a year's wages each to his housekeeper and maids.

DR. HENRY SHACKLETON, of Longton Grove, Sydenham, father of Sir Ernest Shackleton, left estate valued at £306.

MR. W. R. AWDRY, M.B., M.R.C.S., formerly medical officer for the Thornbury Union and the Berkeley Hospital, left estate valued at £23,648.

DR. G. C. SLIMON, of Red House, Mare Street, Hackney, left £11,997.

